# Association of oestrogen receptor beta 2 (ERβ2/ERβcx) with outcome of adjuvant endocrine treatment for primary breast cancer – a retrospective study

**DOI:** 10.1186/1471-2407-7-131

**Published:** 2007-07-18

**Authors:** Raman Vinayagam, D Ross Sibson, Christopher Holcombe, Vijay Aachi, Michael PA Davies

**Affiliations:** 1 Clatterbridge Cancer Research Trust, J.K. Douglas Laboratories, Clatterbridge Hospital, Bebington, Wirral, Merseyside, UK; 2 Division of Surgery and Oncology, University of Liverpool, Liverpool, UK; 3 Breast Services, Linda McCartney Centre, Liverpool and Broadgreen University Hospital NHS Trust, Liverpool, UK; 4 Department of Pathology, Royal Liverpool and Broadgreen University Hospital NHS Trust, Liverpool, UK

## Abstract

**Background:**

Oestrogen receptor beta (ERβ) modulates ERα activity; wild type ERβ (ERβ1) and its splice variants may therefore impact on hormone responsiveness of breast cancer. ERβ2/ERβcx acts as a dominant negative inhibitor of ERα and expression of ERβ2 mRNA has been proposed as a candidate marker for outcome in primary breast cancer following adjuvant endocrine therapy. We therefore now assess ERβ2 protein by immunostaining and mRNA by quantitative RT-PCR in relation to treatment outcome.

**Methods:**

ERβ2-specific immunostaining was quantified in 141 primary breast cancer cases receiving adjuvant endocrine therapy, but no neoadjuvant therapy or adjuvant chemotherapy. The expression of mRNA for ERβ2/ERβcx was measured in 100 cases by quantitative RT-PCR. Statistical analysis of breast cancer relapse and breast cancer survival was performed using Kaplan Meier log-rank tests and Cox's univariate and multivariate survival analysis.

**Results:**

High ERβ2 immunostaining (Allred score >5) and high ERβ2 mRNA levels were independently associated with significantly better outcome across the whole cohort, including both ERα positive and negative cases (Log-Rank P < 0.05). However, only ERβ2 mRNA levels were significantly associated with better outcome in the ERα + subgroup (Log-Rank P = 0.01) and this was independent of grade, size, nodal status and progesterone receptor status (Cox hazard ratio 0.31 P = 0.02 for relapse; 0.17 P = 0.01 for survival). High ERβ2 mRNA was also associated with better outcome in node negative cases (Log Rank P < 0.001).

ERβ2 protein levels were greater in ERα positive cases (T-test P = 0.00001), possibly explaining the association with better outcome. Levels of ERβ2 protein did not correlate ERβ2 mRNA levels, but 34% of cases had both high mRNA and protein and had a significantly better outcome (Log-Rank relapse P < 0.005).

**Conclusion:**

High ERβ2 protein levels were associated with ERα expression. Although most cases with high ERβ2 mRNA had strong ERβ2 immunostaining, mRNA levels but not protein levels were independently predictive of outcome in tamoxifen-treated ERα + tumours. Post-transcriptional control needs to be considered when assessing the biological or clinical importance of ERβ proteins.

## Background

Oestrogen Receptor alpha (ERα) is an accepted prognostic marker in breast cancer and is used to plan adjuvant endocrine treatment (e.g. use of the anti-oestrogen tamoxifen). The majority of the breast cancers are positive for ERα (ERα+), but not all patients with ERα+ cancer respond to endocrine therapy and many subsequently succumb to local relapse or metastasis. The failure of some breast cancers to respond to tamoxifen, currently the most common adjuvant endocrine treatment, is a major clinical problem and several resistance mechanisms have been elucidated [1,2].

ERβ and its splice variants are differentially expressed in a variety of normal tissues and cancers including breast [3,4], but not all published studies are in agreement about the role of ERβ isoforms in breast cancer [4-13]. The ERβ2/ERβcx variant arises from alternative splicing of the last ERβ exon. This produces a truncated ERβ protein unable to bind oestradiol as a result of a disorientated helix12 in the ligand binding domain [14,15]. ERβ2 acts as a dominant negative modulator of ERα [14,16] and therefore might be expected to have a protective effect in breast tumorigenesis or outcome, at least for ERα + breast cancer.

Further verification of ERβ variants, including ERβ2, as potential clinical markers is still required. Many previous studies make use of mRNA levels as a surrogate marker for ERβ protein expression and few have attempted to relate mRNA to protein levels. Other studies that do assess the expression of ERβ protein use techniques that rely on detection of N-terminal epitopes that are shared by most variants. A good proportion of studies also fail to take into account menopausal status, stage of the disease or the treatment given.

We have previously identified ERβ2 mRNA levels as being more closely associated with treatment outcome than mRNA levels of ERβ1 or ERβ5 [17] in a treatment-specific cohort of postmenopausal women receiving adjuvant endocrine treatment but not chemotherapy. However in the same setting, mRNA levels for the wild-type ERβ1 isoform do not correlate well with protein levels [9]. With the aim of clarifying the significance of ERβ2 expression in tamoxifen response and investigating the relationship between ERβ2 mRNA and protein levels, we have therefore set out to evaluate both expression of ERβ2 protein by immunostaining and expression of ERβ2 mRNA by quantitative RTPCR (qRTPCR). Our hypothesis was that ERβ2 may be associated with outcome following adjuvant tamoxifen treatment of breast cancers and therefore be useful as a predictive marker or give some insight into mechanisms of resistance.

We were able to confirm a significant association of both high ERβ2 protein and high mRNA levels with good outcome, but ERβ2 protein levels were not a useful marker of outcome. Strong ERβ2 staining was associated with better outcome, but not independently of ERα. Although ERβ2 mRNA and protein levels did not correlate with each other, approximately one third of cases (34%) were seen to have both high mRNA and high protein levels; these had a significantly better outcome than the other cases, so ERβ2 protein may have a role in improved outcome for a subset of breast cancers.

## Methods

### Patients and specimens

Patients undergoing treatment for invasive breast cancer during the period 1993 and 1999 at the Royal Liverpool University Hospital were identified from a database at the Cancer Tissue Bank Research Centre (CTBRC), University of Liverpool [9]. A total of 141 postmenopausal patients (Table 1) with primary breast cancer were selected, median age was 68 years (range 47–87). They had been treated by surgery (47 mastectomy, 94 wide local excision) and radiotherapy (70 cases), but had not received systemic chemotherapy or primary endocrine therapy. All patients received adjuvant endocrine therapy; either tamoxifen (n = 133) or as part of the ATAC trial (n = 8). Clinical and histological characteristics are summarized in Table 1. ERα and progesterone receptor (PgR) status was obtained from review of histopathology notes or determined immunohistochemically using a cut-off of 10% positive cells [9]. Ki67 immunostaining was reported previously as % positive tumour cells [9]. Clinical follow-up data was recorded by retrospective case-note review with data from surviving patients censored at the date last seen. Outcome measures were breast cancer relapse (BCR) and breast cancer survival (BCS). Median follow-up was 71 months for BCR (range 9 to 113) and 79 months for BCS (range 11 to 113). Ethical approval for the study was obtained from The Liverpool Adult Research Ethics Committee (Reference 01/116), who also approved the collection of samples by the CTBRC with informed consent.

**Table 1:** Patient characteristics

**Characteristic**	**Immunostaining cohort **n (total = 141)	**qRTPCR cohort **n (total = 100)
**Histology**		
invasive ductal	121	85
others	20	15
**Grade**		
G1	22	14
G2	58	44
G3	61	42
**Size**		
T1	63	44
T2	74	53
T3	3	2
unknown	1	1
**Nodal status**		
+	51	39
-	67	49
unknown	23	12
**Vascular invasion**		
present	60	41
absent	81	59
**ERα^1^**		
+	98	70
-	43	30
**PgR^1^**		
+	69	53
-	72	47
**ERβ2 protein**		
high^2^	100	72
low	41	19
**ERβ2 mRNA**		
high^2^	49	34
low	51	30

^1 ^data from O'Neill *et al*. [9]

^2 ^based on ROC-derived cut-points

Based on estimates of proportions of ERβ2 positive cases from our previous study [17] and available outcome data, we determined that this study would have 80% power with an α value of 0.05 to detect a hazard ratio below 0.73 or above 1.40 in the whole cohort (below 0.63 or above 1.74 in the ERα + cohort), which we considered appropriate to give an indication of clinical utility.

### Immunohistochemistry

Histological sections (4 μm) were cut from the formalin-fixed, paraffin-embedded specimens, placed onto 3-aminopropyltriethoxysilane-coated slides and endogenous peroxidase activity was blocked using 3% (v/v) hydrogen peroxide. Tissues were subjected to antigen retrieval by microwaving for 10 minutes in antigen unmasking solution (H3300, Vector Laboratories Ltd., Peterborough, UK). Slides were pre-incubated in Protein Block Serum-Free (DakoCytomation, Ely, UK) for 10 minutes. Immunostaining for ERβ2 was performed overnight at 4°C with mouse anti-human ERβ2 monoclonal antibody MCA2279S (clone no 57/3, Serotec; raised to the unique C-terminal region [18] and previously used for breast tumour staining [4]), diluted 1 in 25 in 0.1% (w/v) BSA, 50 mM Tris, 15 mM NaCl, pH7.6. The bound antibodies were detected using the DAKO LSAB2 system, according to manufacturer's recommendations (DakoCytomation). The bound antibodies were visualized as a brown stain by incubating with DAB chromogen (Sigma-Aldrich, Gillingham, UK). Sections were counterstained with Mayers' Haemotaxylin (Sigma-Aldrich) and mounted in DPX (Merck, Dorset, UK). In controls the ERβ2 antibody was preincubated with a molar excess of immunising synthetic peptide (CMKMETLLPEATMEQ [18]) prior to application to sections from positively-staining specimens. Nuclear staining was abolished in these blocked controls, but some cytoplasmic staining remained. Scoring of tumour sections was performed for nuclear staining only. Stained slides were analysed independently by two observers (RV and VA) using light microscopy; the percentage of positively stained malignant cells was estimated (%+) as was the staining intensity, an immuno-score (Allred) was calculated according to the Allred system [19].

### qRTPCR

RNA of suitable quality for 100 cases was obtained from the CTBRC; testis RNA (Promega, Southhampton, UK) and MCF7 cell line RNA were used as positive controls. Cases were selected for RNA analysis following independent histological review of adjacent sections, so as to avoid high levels of tissue heterogeneity. Samples from all cases consisted of at least 75% tumour cells and 67% of cases had at least 90% tumour cells. Inflammatory infiltrates were present in a minority of cases (at 10% in 15 cases and at 25% in 4 cases).

Reverse transcription was performed in duplicate as described previously with oligo-dT primers [17], but using 1.5 μg total RNA and Superscript III reverse transcriptase (Invitrogen, Paisley, UK). Quantitative PCR for ERβ2 was performed on a Bio-Rad Icycler Real-Time PCR machine (Bio-Rad Laboratories Ltd., Hertfordshire, U.K.) using 4 μ1 of a 1/2 dilution of cDNA per reaction (equivalent to cDNA from approximately 150 ng of total RNA). ERβ2 PCR reactions included 1× IQ Supermix (Bio-Rad) and PCR primers and a Taqman probe (as given in Table 2). For control gene PCR (HPRT, GAPDH) and ERα 4 μl of a 1/50 dilution was used and the reaction contained IQ SYBR Green Supermix (Bio-Rad). The PCR reactions consisted of a hot-start *Taq *Polymerase activation step of 95°C for 3 minutes, followed by conditions shown to be produce unique, specific bands for each mRNA (Table 2). Expression levels of mRNA for each gene were calculated using standard curves produced with the relevant cloned cDNAs and correcting for the control genes (HPRT and GAPDH). All amplicons crossed introns to avoid amplification of genomic DNA and the identity of PCR products was confirmed by agarose gel electrophoresis and DNA sequence analysis, as described previously [17].

**Table 2:** qPCR conditions

**Gene**	**Primers/Probes^3^**	**Conc. **(μM)	**Amplicon**	**Cycling conditions**
**ERβ2^1^**	For-ATCCATGCGCCTGCTAAC	0.5	bases 1265–1343	50 cycles:
	Rev-GAGTGTTTGAGAGGCCTTTTCTG	0.5	GenBank: AF124790	95°C 20s, 52°C 20s
	Probe-TCCTGATGCTCCTGTCCCACGTCA	0.2		
**ERα^2^**	For-CCACCAACCAGTGCACCATT	1.0	bases 1030–1137	40 cycles: 95°C 20s
	Rev-GGTCTTTTCGTATCCCACCTTTC	1.0	GenBank: NM_000125	60°C 20s, 72°C 30s
**HPRT^2^**	For-GTGTTGGATATAAGCCAGACTTTGTT	1.0	bases 597–763	40 cycles:
	Rev-AACTCAACTTGAACTCTCATCTTAGGC	1.0	GenBank: NM_000194	94°C 30s, 64°C 60s
**GAPDH^2^**	For-GCATCCTGGGCTACACTGAG	0.5	bases 917–1079	40 cycles:
	Rev-TCCACCACCCTGTTGCTGTA	0.5	GenBank: NM_002046	94°C 30s, 65°C 90s

^1 ^primers from Critchley *et al. *[25];

^2 ^primers designed in-house.

^3 ^For = forward primer; Rev = reverse primer; Probe = Taqman probe.

### Statistical analysis

Power calculations were performed using the PS program [20] with survival analysis implementation of Schoenfeld and Richter [21]. All other statistical analyses were performed using the SPSS package (Windows, v.11). The degree of agreement for immunostaining between observers was assessed using the Kappa statistic. Pearson correlation and Spearman's rank correlation were used as measures of association. Student's T-test and the Mann-Whitney U test (MW) were used to compare between groups of cases defined by other variables; for paired data paired T-tests and Wilcoxon signed ranks tests were used. Optimal cut-points for continuous variables were determined using Receiver Operating Characteristic (ROC) plots for BCR and BCS at 5 years after surgery. Curves for outcome were generated using the Kaplan-Meier method for censored data, with surviving patients' data being censored at the date of their last clinic visit, and were compared using the Log Rank test. Unadjusted hazard ratios (HR) ± 95% confidence intervals (CI) were obtained using Cox's univariate analysis. Cox's regression model was used for multivariate survival analysis. For outcome analysis grade was dichotomised as grade 3 *vs*. other grades, size was dichotomised as T1 *vs*. other sizes.

## Results and Discussion

Previous assessments of the role of ERβ2 in breast cancer treatment outcome have been limited, with most clinical studies being performed in broader groups of patients and focussing on other associations, largely related to pathology. Our own previous data [17] was based on a semi-quantitative RTPCR analysis using an assay in which ERβ5 is co-amplified with ERβ2 and distinguished based on size of the PCR product, similar to the triple-primer assay used elsewhere [22,23]. We found that, using the arbitrary cut-off imposed by detection sensitivity, ERβ2 mRNA expression was more closely associated with survival benefit than ERβ1 or ERβ5 mRNA expression. We therefore set out to establish whether ERβ2 protein levels similarly predicted patient outcome. We defined discriminatory cut-points of ERβ2 levels in a non-arbitrary manner, using ROC analysis, and used these to assess the relationship between ERβ2 expression and outcome.

### Immunohistochemical staining for ERβ2

**Figure 1:**
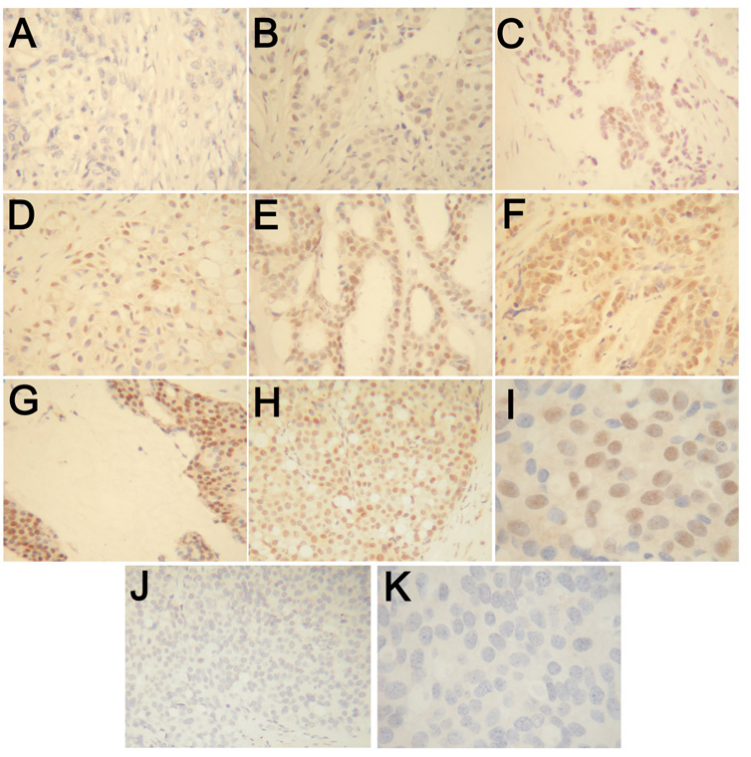
**Immunohistochemical staining for ERβ2**. Breast carcinomas showing different levels of staining; examples of Allred score 0 (A), 3 (B), 4 (C), 5 (D), 6 (E), 7 (F) and 8 (G). H-K are low (H, J) and high (I, K) magnification images of the same tumour stained normally (H, I) and following blocking with synthetic peptide (J, K).

A cohort of 141 cases were stained by immunohistochemistry for ERβ2 (Table 1) including 98 ERα + cases. ERβ2 staining was assessed by 2 observers (R.V., V.A.) using the Allred scoring system and also as percentage positive cells (%+), with good agreement between observers (Allred Spearman 0.91 P = 1.0 × 10^6^, %+ Pearson 0.92 P = 3.4 × 10^59^). At the cut-point used for outcome analysis the Kappa score was 0.87. A consensus score was produced and used herein, representative examples of immunostaining are shown in Figure 1. The frequencies of each score were: score 0, 2 cases (1.4%); 3, 3 cases (2.1%); 4, 9 cases (6.4%); 5, 27 cases (19.1%); 6, 39 cases (27.7%); 7, 61 cases (43.3%).

ERβ2 immunostaining significantly correlated with that for ERα (%+ Pearson 0.42 P = 7.8 × 10^7^, Allred Spearman 0.40 P = 4.1 × 10^6^) and to a lesser extent PgR (%+ Pearson 0.18 P = 0.035). ERβ2 immunostaining was greater in ERα + cases (mean %+ = 69) than in ERα- cases (mean %+ = 52; P = 0.00001 T-test) and ERβ2 Allred score was greater in PgR+ cases than PgR- cases (P = 0.033 MW). The percentage of ERβ2 positive cells were somewhat lower in grade 3 cases (P = 0.042 MW), in keeping with the association with ERα status. There was no association with Ki67 staining, vascular invasion, nodal status, age or size, or with ERβ1-specific immunostaining [9]; most previous studies have similarly failed to show clear links to many clinical and pathological parameters.

The association seen here between ERβ2 and ERα has not always been seen by others. Although case selection and clinical setting may have some bearing on this, it is also possible that such correlations are due to better tissue preservation of antigens in some blocks of tissue. We do not think that this is the case here, as in the same cohort ERα but not ERβ2 inversely correlated with p53 immunostaining (unpublished data) and ERα did not correlate with ERβ1 [9]. If antigen preservation was a major influence on immunostaining patterns it is unlikely that such complex inter-relationships would be evident.

### Association of ERβ2 protein with patient survival

Using the Allred scoring system, tumours were designated as either ERβ2 low (score 5 or lower, n = 39) or ERβ2 high (score 6 or higher, n = 97, 71%). ERβ2 status significantly associated with ERα status (P = 0.001 Chi square) and within the subgroup of ERα positive women who received adjuvant tamoxifen there were 18 ERβ2 low cases and 67 ERβ2 high cases (79%).

**Figure 2:**
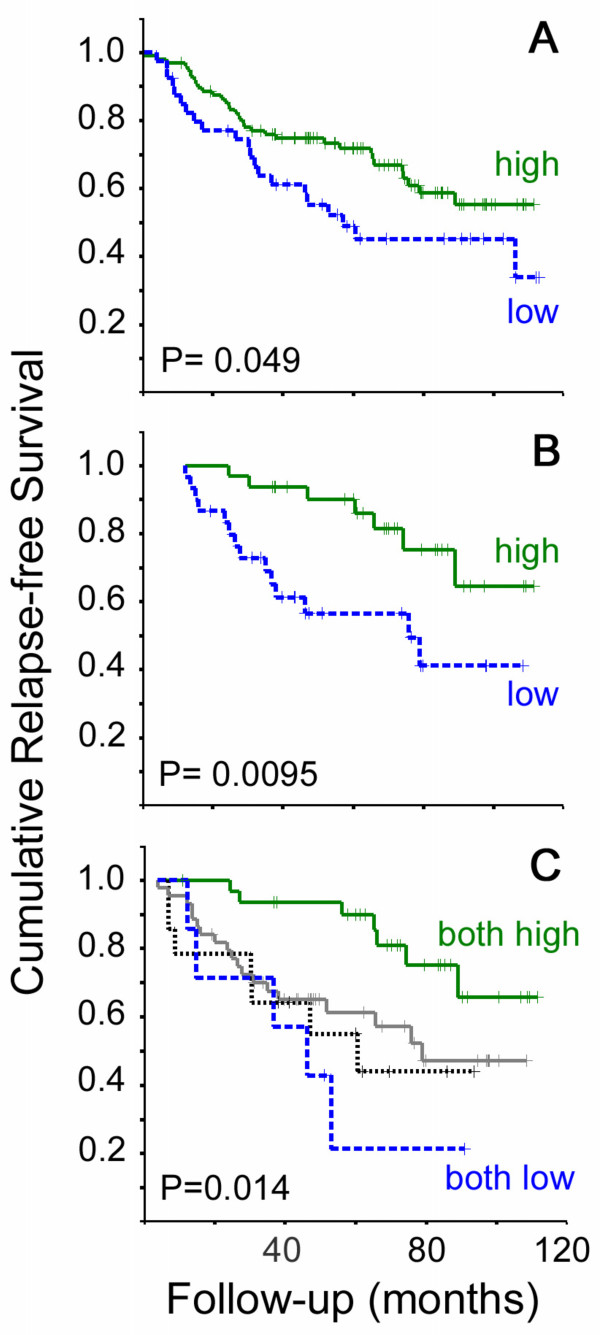
**Kaplan Meier plots for breast cancer relapse**. Plots are shown for dichotomised levels of ERβ2 immunostaining in the whole cohort (A, 34 events in 97 ERβ2 high cases and 21 events in 40 ERβ2 low cases) and dichotomised levels of ERβ2 mRNA in the ERα + tamoxifen-treated cohort and (B, 7 events in 32 ERβ2 high cases and 14 events in 30 ERβ2 low cases). Unbroken green lines represent cases with high levels of ERβ2, dotted blue lines represent cases with low levels. In C cases from the whole cohort were classed as: high for both protein and mRNA (unbroken green line, 7 events in 32 cases); high for protein, low for mRNA (unbroken grey line, 19 events in 44 cases); low for protein, high for mRNA (dotted unbroken black line, 7 events in 14 cases); or low for both protein and mRNA (dotted blue line, 5 events in 7 cases). In all cases crosses represent censored data and P values are given for Log Rank tests.

**Figure 3:**
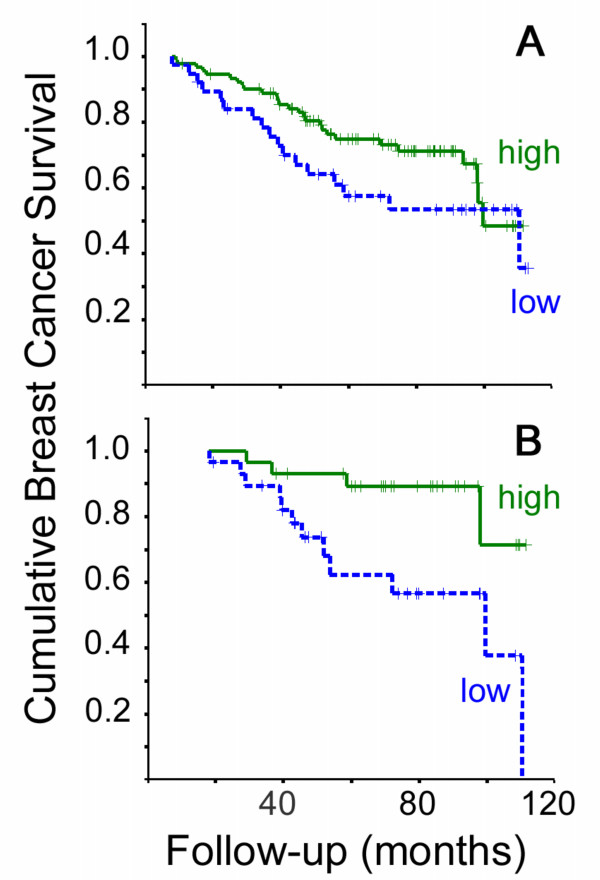
**Kaplan Meier plots for breast cancer survival**. Plots are shown for dichotomised levels of ERβ2 immunostaining in the whole cohort (A, 27 events in 91 ERβ2 high cases and 17 events in 38 ERβ2 low cases) and dichotomised levels of ERβ2 mRNA in the ERα + tamoxifen-treated cohort and (B, 4 events in 29 ERβ2 high cases and 12 events in 29 ERβ2 low cases). Unbroken green lines represent cases with high levels of ERβ2, dotted blue lines represent cases with low levels. In all cases crosses represent censored data and P values are given for Log Rank tests.

Within the group as a whole (ERα + and ERα- cases), high ERβ2 protein levels were significantly related to a better relapse free survival (BCR P = 0.049 Log Rank, Figure 2), but not breast cancer survival (BCS P = 0.16, Figure 3). However, in both cases the survival curves converge at later time-points; with shorter follow-up time a stronger relationship with outcome was seen (5-year BCR P = 0.018 Log Rank, HR 0.50 CI 0.27–0.90 P = 0.020; 7 year BCS P = 0.048 Log Rank, HR 0.50 CI 0.27–0.90 P = 0.020).

When ERα status and ERβ2 immunoscore were included as the only two variables in multivariate analysis of 5 year BCR, ERα status was independently significant (HR 0.38 CI 0.22–0.66 P = 0.001) whereas ERβ2 did not retain independent significance (HR 0.76 CI 0.43–1.33 P = 0.33). When ERβ2 immunoscore, ERα, grade, size and nodal status were included in this multivariate analysis, only nodal status (HR 3.1 CI 1.5–6.2 P = 0.001) and grade (HR 1.5 CI 1.1–2.2 P = 0.026) were independently significant. When considering only ERα +, tamoxifen-treated cases there was no relationship between ERβ2 immunostaining at outcome (BCR P = 0.95, BCS P = 0.65 Log Rank).

One previous study of only 50 ERα positive cases using immunostaining with a different antibody raised to the same ERβ2-specific epitope [7] similarly failed to show any predictive association with adjuvant tamoxifen treatment. However this analysis was based on detecting differences in staining between "sensitive" and "resistant" cases using the crude measure of relapse within 5 years of tamoxifen therapy. Unpublished observations [12] also failed to show any predictive value in an adjuvant setting and a similar lack of association between ERβ2 immunostaining and outcome has recently been demonstrated in the neoadjuvant setting [4]. Hence the early outcome benefit seen with strong ERβ2 immunostaining was not identified previously. However, an association of ERβ2 protein with a favourable outcome has been seen in a metastatic and locally advanced setting [10]. In this case, not only was the clinical setting different, but ERβ2 was assessed by western blot. Therefore the present study is the largest to date to assess immunostaining of ERβ2 as a predictive marker of outcome in the postmenopausal, adjuvant endocrine setting. Results indicate that ERβ2 protein levels did not apparently relate closely to outcome for ERα + cases. Rather there was some association of ERβ2 immunostaining with better outcome in broader cohorts of patients (including ERα- cases), due in part to a correlation between ERα and ERβ2 protein levels.

### Association of ERβ2 mRNA with patient survival

ERβ2 immunostaining results are at odds with previous semi-quantitative RTPCR results. We therefore performed a repeat RTPCR analysis on a larger series of patients, but with fully quantitative RTPCR using independent cDNA synthesis reactions and different splice variant specific PCR conditions. A subgroup of 100 cases (Table 1) with suitable quality mRNA available were used in qRTPCR for ERβ2, ERα and control genes HPRT and GAPDH. Expression of ERβ2 mRNA (mean 0.006 attomoles per μg total RNA) was significantly lower (P < 10^6 ^paired T-test) than that of ERα (mean 25 attomoles per μg total RNA). These low levels of ERβ mRNA (also seen with ERβ1 and ERβ5, results not shown) may contribute to technical difficulties in reproducibly measuring ERβ variants and hence to the lack of consistency between different studies.

In the 100 case (ERα + and ERα -) qRTPCR cohort (Table 1), high grade (BCR & BCS P ≤ 0.001), positive nodal status (BCR & BCS P ≤ 0.0005), larger size (BCS P = 0.042), ERα negative status (BCR P = 0.009, BCS P = 0.041) and PgR negative status (BCR P = 0.032, BCS P = 0.026) were all associated with poor outcome (Log Rank). Using an ROC-derived optimal cut-point (0.0040 attomoles per μg total RNA) for this 100 case cohort in Kaplan Meier Log Rank analysis, there was a significant association between higher ERβ2 mRNA expression and good outcome (BCR P = 0.046 Log Rank, HR 0.51 CI 0.26–1.00 P = 0.0496). As with ERβ2 immunoscore, this association was stronger at 5 years (5-year BCR P = 0.016 Log Rank, HR 0.39 CI 0.18–0.87 P = 0.020). Notably, unlike ERβ2 immunostaining, no significant association was found between ERβ2 mRNA and ERα immunostaining. Also unlike ERβ2 protein, ERβ2 mRNA and ERα status were independently associated with BCR at 5 years (ERβ2 HR 0.43 CI 0.20–0.95 P = 0.036, ERα HR 0.36 CI 0.18–0.75 P = 0.006). These two measures of ERβ2 expression therefore seem to behave differently in relation to ERα status and treatment outcome.

Further outcome analysis was limited to ERα positive women who received adjuvant tamoxifen and had a defined breast cancer related outcome (n = 62 BCR, n = 58 BCS). High grade (BCR P = 0.006, BCS P = 0.0008) and positive nodal status (BCR P = 0.003, BCS P = 0.007) maintained their association with worse outcome (Log Rank). ROC plots for BCR and BCS at 5 years indicated a significant relationship between good outcome and high qRTPCR values for ERβ2 (BCR area under curve 0.68 CI 0.52–0.84, P = 0.036) and the optimal cut-point was 0.0039 attomoles per μg total RNA. There were significant associations between outcome and ERβ2 mRNA level using the ROC-derived cut-point (Figures 2 and 3). High ERβ2 mRNA was significantly associated with better outcome (BCR P = 0.0095 Log Rank, HR 0.32 CI 0.13–0.79; BCS P = 0.011 Log Rank, HR 0.25 CI 0.08–0.79). The 5-year cumulative relapse-free population was 81% in the ERβ2-high group (standard error 8%), compared to 55% in the ERβ2-low group (standard error 10%); the 5-year cumulative BCS was 89% in the ERβ2-high group (standard error 6%), compared to 62% in the ERβ2-low group (standard error 10%).

In Cox multivariate analysis of the ERα + tamoxifen-treated cohort including grade, size, nodal status and PgR status, high ERβ2 mRNA had independent significance for good outcome: for BCR ERβ2 (HR 0.31 CI 0.11–0.86, P = 0.024) and nodal status (HR 3.7 CI 1.2–11.5, P = 0.022) were independently significant; for BCS ERβ2 (HR 0.17 CI 0.05–0.65, P = 0.0095) and grade (HR 1.8 CI 1.03–3.3, P = 0.041) were independently significant. Notably there was no significant association between ERβ2 and grade, size, nodal status or PgR status in this treatment-specific cohort (all P > 0.35 Chi-square). In ERα +, node negative cases (n = 33), using a lower cut-off (0.00185 attomoles per μg total RNA), ERβ2 was significantly associated with better outcome (BCR P = 0.0005, BCS P < 0.00005 Log Rank); the 5 year cumulative relapse-free population was 96% in the ERβ2-high group (standard error 4%), compared to 39% in the ERβ2-low group (standard error 24%).

Our results indicate that ERβ2 isoform mRNAs may be an independent marker for ERα + cases that respond well to adjuvant tamoxifen treatment. In node negative cases, where the need for additional markers of response is greatest, our study shows that low ERβ2 mRNA levels are significantly related to worse outcome; as the cases in this subgroup analysis was small, a larger study of node negative patients is warranted. The fully quantitative nature of the qRTPCR results allows comparison of mRNA levels between different ER isoforms and of variant levels between tumours, but necessitated selection of optimal cut-points (in this case using ROC analysis) for the dichotomization required for standard outcome analysis. It should be noted that, whilst such dichotomization is useful in demonstrating associations with outcome, true utility of ERβ variant mRNA measurement will only be demonstrated with larger patient cohorts and may be better achieved by treating mRNA quantitation as a continuous variable, as in other RTPCR based outcome predictors [24].

### Association of staining for ERβ2 protein with mRNA expression

Associations between high levels of ERβ2 protein (immunoscore) or mRNA (qRTPCR) and improved outcome have been seen, but only the qRTPCR results are significant in the clinically relevant ERα + cohort. It is therefore important to establish the relationship between mRNA and protein levels in clinical samples. Notably, most previous RTPCR-based analyses have failed to take into account the possible translational control when assigning biological or clinical relevance to ERβ isoform expression.

When assessing the relationship between immunostaining and qRTPCR for paired samples from each case, no correlation was seen between levels of protein and mRNA for ERβ2 [Pearson (%+) -0.12 P = 0.24; and Spearman (Allred) -0.08 P = 0.40]. This is in contrast to ERα in the same cohort [Pearson (%+) 0.30 P = 0.003; Spearman (Allred) 0.50 P = 1.0 × 10^-6^], but a similar lack of correlation was seen previously for ERβ1 [9]. Due to tissue heterogeneity, any mRNA analysis of tissue homogenates without selection can contribute to discordance with immunostaining results that are scored on specific cell types. In order to minimise the impact of such artefacts, we selected cases for mRNA analysis that had high proportions of tumour cells (see Methods). It is known that lymphocytes express ERβ2 mRNA, but when 14 cases with inflammatory infiltrates were excluded there was still no significant correlation between ERβ2 mRNA and protein expression. A major factor in the discordance is that many cases express high levels of protein, but low mRNA levels; a situation that is not likely to arise from expression of mRNA in non-tumour cells. It is however possible that heterogeneity of expression in the different parts of the tumour specimen used for mRNA and protein analysis contributes to the lack of correlation and *in situ *analysis of mRNA and protein in adjacent sections might address this.

High ERβ2 protein levels and high ERβ2 mRNA levels, when entered into a Cox multivariate model, were independently associated with better relapse-free survival in the whole cohort (ERβ2 protein HR 0.40 CI 0.20–0.80 P = 0.010, ERβ2 mRNA HR 0.43 CI 0.22–0.83 P = 0.013). In multivariate analysis of mRNA and protein in the ERα + tamoxifen-treated cohort, only high ERβ2 mRNA levels were significantly associated with lower BCR (HR 0.28 CI 0.212–0.72 P = 0.008), but a trend remained for protein (HR 0.42 CI 0.15–1.19 P = 0.10). Similar results were obtained for analysis of BCS. This indicates that both mRNA and protein levels may contribute to the relationship of ERβ2 with improved outcome.

Using the cut-points optimised for outcome analysis, the majority of cases (69%) with high ERβ2 mRNA levels also had high levels of ERβ2 protein. However, only a minority of cases (44%) with high ERβ2 protein were also classified as having high ERβ2 mRNA. Thus ERβ2 mRNA expression is frequently associated with expression of significant levels of ERβ2 protein, but ERβ2 protein expression is often dissociated from mRNA expression. Hence, there is a subset of cases (34%) with concomitant high ERβ2 mRNA and protein and another subset of cases (44%) in which high protein levels are not accompanied by high mRNA levels. The cases with both high ERβ2 protein and mRNA had a significantly better outcome than those with low levels of either mRNA or protein or both (P = 0.011 Log Rank, Figure 2). When cases with high ERβ2 protein and RNA were compared a group consisting of all other cases they had significantly better outcome: in the whole cohort of ERα + and ERα- cases, (BCR P = 0.002 Log Rank, HR 0.67 CI 0.51–0.88 P = 0.004; BCS P = 0.003 Log Rank, HR 0.61 CI 0.43–0.87 P = 0.006) and for ERα + tamoxifen-treated cases (BCR P = 0.004 Log Rank, HR 0.61 CI 0.43–0.88 P = 0.009; BCS P = 0.009 Log Rank, HR 0.56 CI 0.34–0.91 P = 0.020). The outcome benefit of concomitant high ERβ2 mRNA and protein levels was particularly marked at shorter follow-up, where this measure was the only independent marker of improved outcome in the ERα + tamoxifen-treated cohort using Cox multivariate analysis including grade, size PgR status and nodal status (5-year BCR HR 0.48 CI 0.24–0.95 P = 0.036, 7 year BCS HR 0.46 CI 0.23–0.92 P = 0.029). In the ERα + tamoxifen-treated, node negative cases, having both high ERβ2 mRNA and protein was significantly related to an improved BCS (P = 0.028 Log Rank).

Although ERβ2 protein levels are apparently not directly related to mRNA levels, expression of ERβ2 protein may be important because good outcome was observed for those cases assessed as having both high mRNA and protein levels and this was independent in multivariate analysis. It is possible therefore that the relatively poor utility of ERβ2 protein assessment by immunostaining as a measure of outcome prediction may be due to high levels of ERβ2 protein in some cases (with lower levels of ERβ2 mRNA) being related to some form of protein stabilization, or detection of inactive ERβ2. The disparity between protein and RNA expression for ERβ2 is even suggestive of an inverse relationship. Nevertheless a significant proportion of cancers (34%) had both high protein and high mRNA levels and these had a significantly better outcome than the remaining cases. This suggests that transcription of ERβ2 mRNA drives ERβ2 protein levels in some cases, and these cases do particularly well on tamoxifen treatment. It is perhaps unsurprising that previous studies of ERβ2 protein expression did not find significant associations between ERβ2 and outcome in ERα + tamoxifen treated cases as these did not include measurement of ERβ2 mRNA levels. They were thus unable to distinguish between ERB2 protein associated with increased transcription and that possibly present due to some form of post-transcriptional control (or perhaps the breakdown of normal control).

## Conclusion

Whilst our data would suggest that high ERβ2 levels could contribute to an improved outcome in a subgroup of patients, it provides further evidence that determination of ERβ2 protein by immunostaining is unlikely to provide the predictive test that is needed for better targeting of additional therapy in those women for whom adjuvant tamoxifen is not likely to be sufficient. The failure to link protein expression to outcome measures does not preclude the use of ERβ2 mRNA levels in a clinical setting. Low ERβ2 mRNA was significantly associated with worse outcomes in ERα + tamoxifen-treated patients independently of other factors such as grade and nodal status. Larger trials to validate ERβ2 mRNA as a biomarker are needed and should be extended to alternative adjuvant endocrine therapies such as aromatase inhibitors.

## List of abbreviations

BCR = breast cancer relapse; BCS = breast cancer survival; CI = confidence interval; ERα = oestrogen receptor alpha; ERβ = oestrogen receptor beta; HR = hazard ratio; MW = Mann-Whitney U test; %+ = percentage positive cells; PgR = progesterone receptor; qRTPCR = quantitative reverse-transcription PCR analysis; ROC = Receiver Operating Characteristic.

## Competing interests

The author(s) declare that they have no competing interests.

## Authors' contributions

RV performed qRTPCR analysis and immunostaining under the supervision of MD. RV and VA scored the immunostaining and MD and RV performed the statistical analysis. MD, DRS and CH participated in conception, design and coordination of the study. All authors helped to draft the manuscript and approved the final version.

## Pre-publication history

The pre-publication history for this paper can be accessed here:


